# Characterization and Comparative Analysis of Chloroplast Genomes in Five *Uncaria* Species Endemic to China

**DOI:** 10.3390/ijms231911617

**Published:** 2022-10-01

**Authors:** Min-Min Chen, Miao Zhang, Zong-Suo Liang, Qiu-Ling He

**Affiliations:** 1 Key Laboratory of Plant Secondary Metabolism and Regulation of Zhejiang Province, College of Life Sciences and Medicine, Zhejiang Sci-Tech University, Hangzhou 310018, China; 2 Shaoxing Academy of Biomedicine, Zhejiang Sci-Tech University, Shaoxing 312366, China

**Keywords:** *Uncaria*, chloroplast genome, comparative analysis, phylogeny

## Abstract

*Uncaria*, a perennial vine from the Rubiaceae family, is a typical Chinese traditional medicine. Currently, uncertainty exists over the *Uncaria* genus’ evolutionary relationships and germplasm identification. The complete chloroplast genomes of four *Uncaria* species mentioned in the Chinese Pharmacopoeia and *Uncaria scandens* (an easily confused counterfeit) were sequenced and annotated. The findings demonstrated that the whole chloroplast genome of *Uncaria* genus is 153,780–155,138 bp in full length, encoding a total of 128–131 genes, containing 83–86 protein-coding genes, eight rRNAs and 37 tRNAs. These regions, which include eleven highly variable loci and 31–49 SSRs, can be used to create significant molecular markers for the *Uncaria* genus. The phylogenetic tree was constructed according to protein-coding genes and the whole chloroplast genome sequences of five *Uncaria* species using four methods. The topology of the two phylogenetic trees showed no difference. The sequences of *U. rhynchophylla* and *U. scandens* are clustered in one group, while the *U. hirsuta* and *U. macrophylla* are clustered in another group. *U. sessilifructus* is clustered together with the above two small clades. New insights on the relationship were revealed via phylogenetic research in five *Uncaria* species. This study will provide a theoretical basis for identifying *U. rhynchophylla* and its counterfeits, as well as the species of the *Uncaria* genus. This research provides the initial chloroplast genome report of *Uncaria*, contributes to elucidating the chloroplast genome evolution of *Uncaria* in China.

## 1. Introduction

The Rubiaceae is the fifth largest flowering plant family spread worldwide. *Uncaria*, a perennial vine from the Rubiaceae family, often climbs on top of other plants with its hook-like inflorescence stalk. It is indigenous to tropical areas of Asia, South America, and Africa and is mainly used for medicinal purposes and as a natural dye. The Pharmacopoeia of the People’s Republic of China contains five species, including *Uncaria sessilifructus, Uncaria sinensis, Uncaria hirsuta, Uncaria macrophylla,* and *Uncaria rhynchophylla*. In China, they are mainly distributed in Yunnan, Guangdong, Guangxi, and southeast Guizhou Provinces [1]. Wild resources often grow in mountain forests or hilly areas. There are differences in agronomic characters, morphological characteristics, effective components, and molecular characteristics among different germplasms.

The *Uncaria* plant contains various of chemical constituents, including alkaloids, flavonoids, triterpenes, aliphatic compounds, phenolic acids, coumarins, lignans, steroids, and anthraquinones [2,3,4,5,6,7]. Alkaloids are the main medicinal components in *Uncaria* spp. Their main types are monoterpene indole alkaloids and β-carboline. Previous studies have shown that rhynchophylline can regulate the expression of phosphoinositide-3-kinase (PI3Ks), protein kinase B and glycogen synthase kinase 3β, which are involved in regulating neuronal signaling pathways [8]. The racemic analogues of rhynchophylline (G2) and their stereoisomers can effectively treat microvascular dysfunction caused by diabetes. In addition, it is reported that isorhynchophylline can cause an effect by regulating multiple signal cascades in hepatocellular carcinoma cells [9].

Chloroplasts are semiautonomous organelles commonly found in higher plants, algae cells, and some protozoa. For photosynthesis and energy transformation in plants, chloroplasts are crucial structures. Plant chloroplast genes are usually composed of photosynthesis, transcriptional translation and expression, other protein-coding genes, and open reading frame (ORF) [10]. The chloroplast genome usually presents a four-segment structure, which is composed of two reverse repeat regions (IR), a large single copy region (LSC), and a small single copy region (SSC) [11]. Two IR regions separate the LSC region from the SSC. Compared to mitochondrial and nuclear genomes, chloroplast genomes have uniparental inheritance, a low rate of nucleotide substitution, and a simple structural design [12,13]. The matrilineal inheritance of the chloroplast genome is responsible for maintaining a relatively stable genetic structure with a rate of evolution intermediate to that of the nuclear and mitochondrial genomes. Therefore, the chloroplast genomes become an ideal resource and a versatile tool for phylogenetic studies at different levels for species identification [14]. However, the chloroplast genomes of *Uncaria* spp. had not been reported, leading to limitations in the mining of its genetic information and the development of phylogenetic studies.

Therefore, in this study, morphological characteristics and the chloroplast genome of four species of *Uncaria* spp. were first reported. A comparative genomic analysis among five species of *Uncaria* was performed to reconstruct the phylogeny and explore the high variability sites to provide pertinent details for potential molecular markers and lay the theoretical foundation for the identification of genuine and counterfeit herbs of *Uncaria*.

## 2. Results

### 2.1. Morphological Comparison

**Figure 1:**
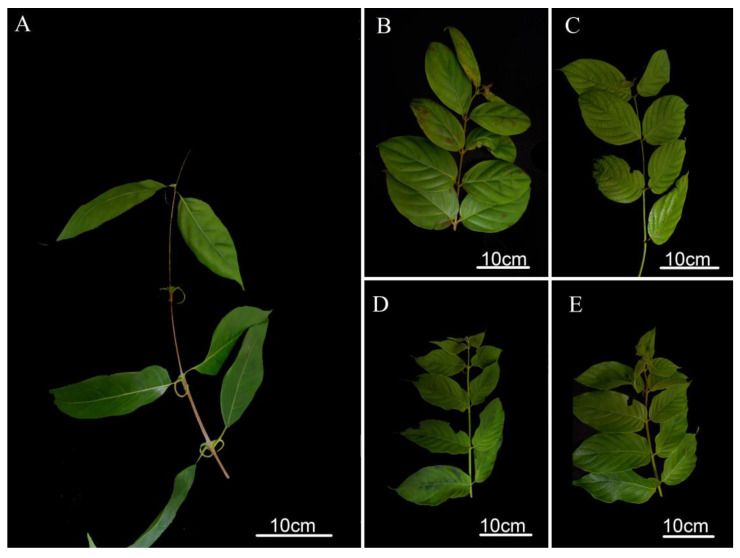
The leaf characteristics of five species in Uncaria. (**A**): *U. rhynchophylla*; (**B**): *U. sessilifructus*; (**C**): *U. macrophylla*; (**D**): *U. hirsuta*; (**E**): *U. scandens*.

The five species of *Uncaria* used in this study are as follows: *Uncaria rhynchophylla, Uncaria macrophylla, Uncaria hirsuta, Uncaria scandens,* and *Uncaria sessilifructus*. The morphological study showed that the stipules of *U. rhynchophylla* were significantly different from the other four species of *Uncaria* plant, which were bifid linear stipules. The stipules of *U. macrophylla, U. hirsuta,* and *U. sessilifructus* are all deeply bifid triangular lobes, while the *U. sessilifructus*’ are slenderer. Furthermore, the size of stipules in *U. scandens* are larger than other species, with which broad strips or triangular lobes. The leaves of the five species of *Uncaria* are all simple and opposite leaves with cylindrical to square stems. The stem epidermal color of *U. macrophylla* and *U. scandens* showed yellowish green while *U. rhynchophylla*, *U. sessilifructus*, and *U. hirsuta* showed lavender to brown, yellow, and light gray to yellowish green colors, respectively. In addition, the leaf characteristics of these five species of *Uncaria* also show marked differences. The leaves of *U. sessilifructus* are leathery, smooth, and brittle, ovate to long elliptic, with gray abaxially. The leaves of *U. scandens* are subpapery and pilose on both surfaces. The leaves of *U. hirsuta* are ovate-lanceolate, almost glabrous on the leaf surface, with a sparse hirsute abaxial side. The leaves of *U. macrophylla* are subleathery, brittle, with hairs only on the veins, and the leaves color is yellowish brown with hirsute abaxially. The leaves of *U. rhynchophylla* are smooth and subleathery, and the surface of young leaves is reddish brown (Figure 1 and Figure S1, Supplementary Table S1).

### 2.2. Chloroplast Genome Sequencing and Assembly

**Figure 2:**
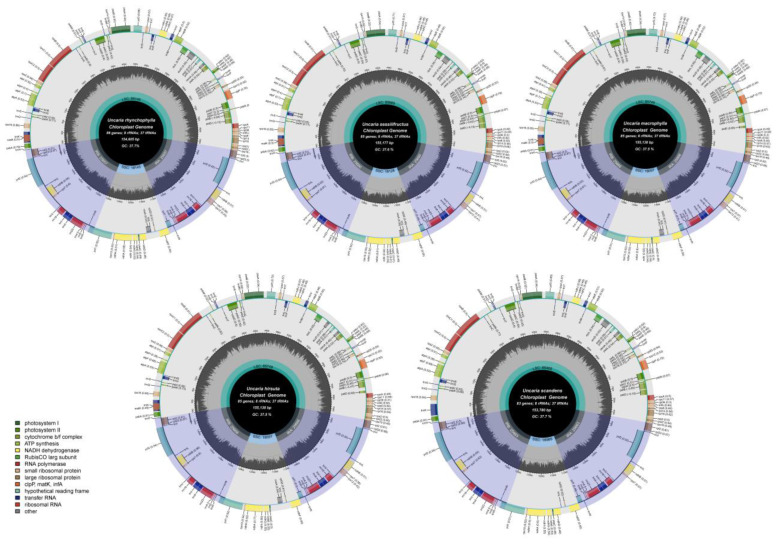
The complete chloroplast maps of five species in *Uncaria*. The gene in the circle is transcribed clockwise while the gene outside is transcribed counterclockwise. Genes are color-coded according to their roles. The AT content is shown in the lighter grey, while the GC content is shown in the deeper grey in the center circle.

Illumina paired-end (150 bp) sequencing was used to obtain the raw reads of *U. sessilifructus, U. macrophylla, U. hirsuta,* and *U. scandens*. All raw reads were processed to eliminate adaptor sequences, short reads, and low-quality bases. Then, 13,659,533–18,093,602 bp clean reads were yielded. Contigs were produced by the de novo assembly, which were subsequently joined to generate the final cp genome for four species. The lengths of chloroplast genome sequences were 154,605, 155,177, 155,138, 155,138, and 153,780 bp for *U. rhynchophylla* (MN723865), *U. sessilifructus, U. macrophylla, U. hirsuta,* and *U. scandens,* respectively (Table 1, Figure 2). Four Uncaria species of chloroplast genome sequences were uploaded to NCBI GenBank.

### 2.3. Chloroplast Genome Structure and Characteristics Analyses

The entire cp genomes showed the standard quadripartite structure found in most angiosperms, which includes the large single copy (LSC), the small single copy (SSC), and two inverted repeats (Ira and Irb) [15]. The length varied from 85,146 bp to 85,749 bp in the LSC region, from 16,989 bp to 18,145 bp in the SSC region, and from 25,657 bp to 25,690 bp in IR region. The percentages of the cp genome encoding protein, rRNAs, and tRNAs are, respectively, 49.88–52.08, 5.83–5.88, and 1.77–1.80, and the remaining 40.27–42.47% is composed of non-coding regions. In all five species, the overall GC content was 37.5–37.7%. The GC contents of the LSC, SSC, and IR regions were 35.4–35.6%, 31.5–31.7%, and 43.2%. The GC contents of IR region were significantly higher than LSC and SSC. It is highest in the IR regions, lowest in the SSC regions and moderate in the LSC regions. The GC contents of the protein-coding genes, rRNA and tRNA were 37.9–38.1%, 55.3–55.4%, and 53.3–53.5%. Meanwhile, the genomes of five *Uncaria* species contained 128–131 genes in total, including 83-86 protein-coding genes, 37 rRNA genes, and eight tRNA genes (Table 2).

In each *Uncaria* species, there were sixteen genes with two copies, which were comprised of five protein-coding genes (*ndhB, rps7, ycf2, rpl2, rpl23*), seven tRNA genes (*trnI-GAU, trnA-UGC, trnL-CAA, trnI-CAU, trnR-ACG, trnV-GAC, trnN-GUU*), and four rRNA genes (*rrn16, rrn23, rrn4.5, rrn5*). The three genes *(clpP, rps12 and ycf3)* had two introns, while nine protein-coding genes (*atpF, rpl2, ndhB, ndhA, rps16, rpoC1, petD, petB, rpl16*) and seven tRNA genes (*trnI-GAU, trnG-UCC, trnV-UAC, trnK-UUU, trnL-UAA, trnL-UAG, trnA-UGC*) only had one intron (Supplementary Table S2).

### 2.4. Codon Usage Analyses

**Figure 3:**
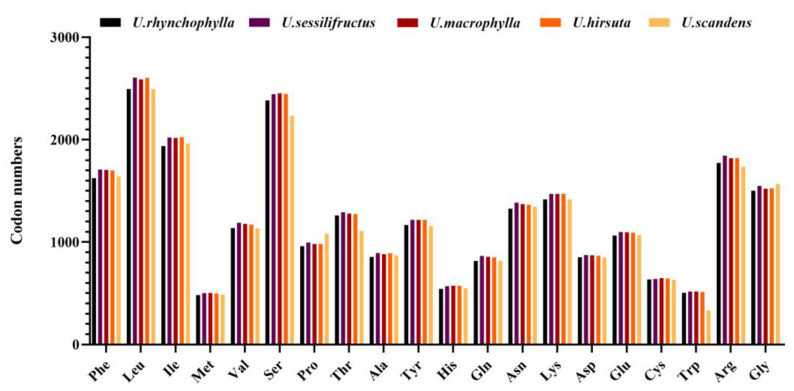
Amino acids and stop codons of codon numbers for the protein-coding regions in five *Uncaria* species. The histogram from left to right is *U. rhynchophylla, U. sessilifructus, U. macrophylla, U. hirsuta,* and *U. scandens*.

We evaluated the codon usage of the protein-coding sequences in five species (Supplementary Table S3). “Count” represents the number of codons in one sample (used universal genetic code) [16]. There were 25,728 (*U. rhynchophylla*), 26,722 (*U. sessilifructus*), 26,578 (*U. macrophylla*), 26,562 (*U. hirsuta*), and 25,699 (*U. scandens*) counts, respectively. Except for the three stop codons, with an average of 1048.6 (varied from 1014 to 1069), the UUU codons for the amino acid phenylalanine (Phe) were the most numerous, while the CGC codon corresponding to arginine (Arg) was the smallest with an average of 116.6 (varied from 112 to 121). With the exception of the unique codons (Trp, Met), Leucine (Leu) was the amino acid with the most codons, with an average number of 2585, while cysteine (Cys) was the least (640.6) (Figure 3).

**Figure 4:**
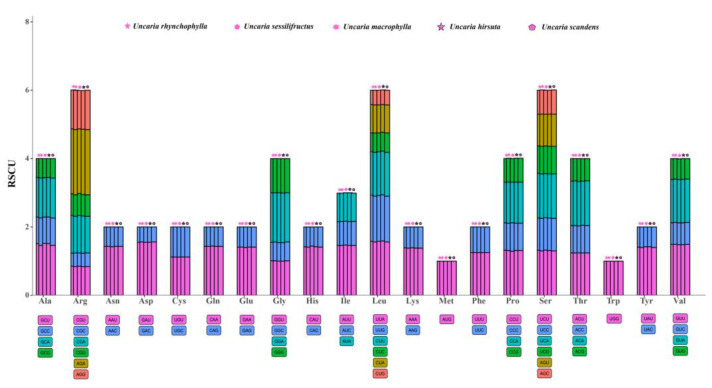
RSCU value comparison plots for amino acids in the protein-coding regions of five *Uncaria* species. The codon colors and the histogram colors match each other, their heights represent the RSCU value. The histogram from left to right is *U. rhynchophylla, U. sessilifructus, U. macrophylla, U. hirsuta,* and *U. scandens*.

In addition, relative synonymous codon usage (RSCU) values are the relationship between the number of practical codon emergence and the number of anticipated codon emergence [17]. If the RSCU > 1, which means that this codon has the higher preference. The results showed that compared with A/T (U), the codons ending with C/G had fewer codons and RSCU < 1. In all protein-coding sequences of five species, 30 (46.875%) codons had RSCU > 1 (higher preference). Most of these codons had an ending of U or A. AGA codon corresponding to the amino acid Arginine (Arg) was the highest preferred across the studied five species with an average RSCU of 1.906. Methionine (Met) and threonine (Thr) had no bias (RSCU = 1). However, 32 (50.00%) codons had RSCU < 1 and low preference (Figure 4).

### 2.5. Repeat Sequence Analysis

**Figure 5:**
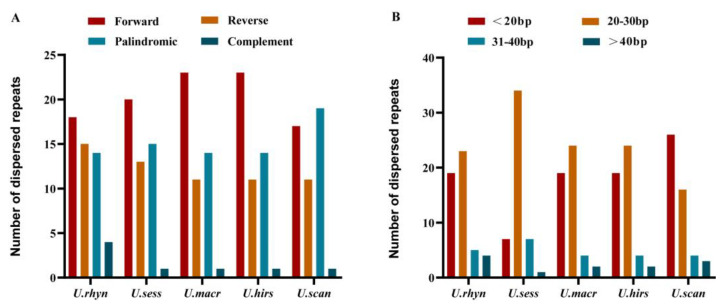
Dispersed repeated sequences analyses in cp genomes for five *Uncaria* species. (**A**) Statistics of four types of dispersed repeated sequences in five cp genomes; (**B**) Number of the dispersed repeated sequences with different period sizes.

Repeat sequences occupy an important position in phylogenetic studies and genome rearrangements [18]. Repeat sequences are indispensable to make indels and substitutions [19]. Dispersed repeats were identified for the five cp genomes using REPuter, including forward repeats (F), reverse repeats (R), palindromic repeats (P), and complement repeats (C). The five *Uncaria* cp genomes contained 49–51 dispersed repeats, comprising 17–23 forward repeats, 14–19 palindromic repeats, 11–15 reverse repeats, and one-four complement repeat. Five cp genomes possessed four types of dispersed repeated sequences. Forward repeat was the most universal type (Figure 5A). Meanwhile, it largely consisted of dispersed repeats with a unit length of <20 bp and 20–30 bp in all five cp genomes (Figure 5B).

**Figure 6:**
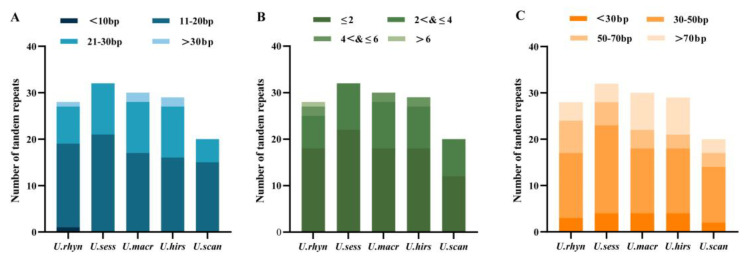
Tandem repeated sequence analyses for five *Uncaria* species in cp genomes. The number of tandem repeats with various period sizes, copy counts, and sequence lengths are shown in (**A**,**B**,**C**), respectively.

Tandem repeats finder was used to identify the tandem repeated sequences for the five cp genomes. The number of the tandem repeated sequences were different in five cp genomes, as follows: 28, 32, 30, 29, 20. It ranged from 20 to 32. *U. sessilifructus* had the most tandem repeats and *U. scandens* had the least. The period size (repeat unit size) for the tandem repeats ranged from 3 to 49 bp. Tandem repeated sequences with periods ranging from 11 to 30 bp predominated in the cp genomes (Figure 6A). The majority of the tandem repeats in the five cp genomes had a copy number of no more than 4 (Figure 6B). Five species’ sequence lengths of tandem ranged from 25 to 98 bp. Tandem repeats with sequence lengths ranging from 30 to 50 bp predominated in all cp genomes (Figure 6C).

### 2.6. SSR Analysis

**Figure 7:**
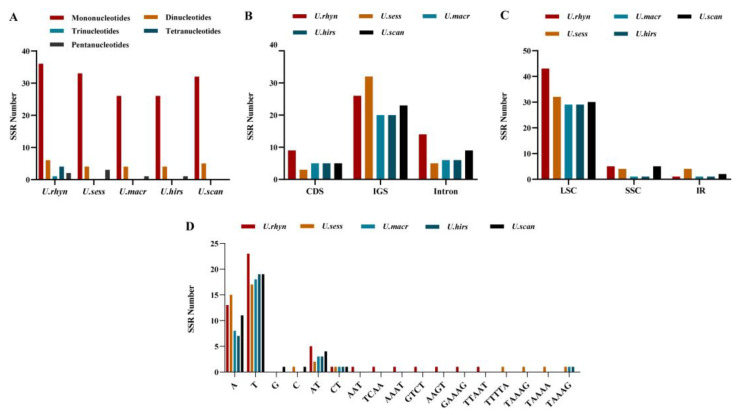
SSR analyses for five *Uncaria* species in cp genomes. (**A**) Number of different SSR types; (**B**) Frequency of identified SSRs in LSC, SSC, and IR regions; (**C**) Frequency of identified SSRs in CDS, IGS (intergenic spacer region), and intron; (**D**) Frequency of identified SSR in different repeat class types.

The five cp genomes’ SSRs were discovered using MISA. There were 49, 40, 31, 31, and 37 SSRs in *U. rhynchophylla, U. sessilifructus, U. macrophylla, U. hirsuta,* and *U. scandens,* respectively. Most SSRs possess a mononucleotide repeat motif in five cp genomes, ranging from 26 to 36. Dinucleotides SSRs are the second most common. The five cp genomes did not have hexanucleotide SSRs. Only *U. scandens* did not find pentanucleotides SSRs. Meanwhile, no trinucleotides SSRs and tetranucleotides SSRs were discovered in *U. sessilifructus, U. macrophylla, U. hirsuta,* and *U. scandens* (Figure 7A).

Five cp genomes yielded a total of 188 SSRs, of which 121 (64.36%) were in IGS, 27 (14.36%) were in CDS, and 40 (21.28%) were in intron. Most loci were located in intergenic spacer (IGS) regions, and the fewest loci were located in CDS, probably due to the IGS regions having higher mutation rates than coding regions. In LSC, IR, and SSC regions, 163 (86.70%), 9 (4.79%), and 16 (8.51%) SSRs were found, respectively. Most SSRs existed in the LSC region (Figure 7BC). In five cp genomes, the mononucleotide A/T repeat units had the highest percentage occupancy (73.47–83.87%). Furthermore, the largest number of mononucleotides SSRs (A/T) was found in *U. rhynchophylla* (13A + 23T), while *U. macrophylla* and *U. hirsuta* had the least mononucleotide SSRs (8A + 18T, 7A + 19T) (Figure 7D).

### 2.7. Contraction and Expansion of Inverted Repeats

**Figure 8:**
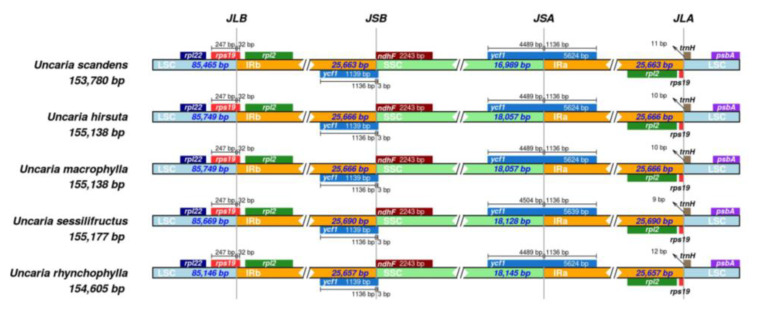
Comparison of LSC, IR, and SSC junction positions among five *Uncaria* species in cp genomes. JLB denotes the LSC/IRb junction, JSB denotes the SSC/IRb junction, JSA denotes the SSC/IRa junction, and JLA denotes the LSC/IRa junction.

**Figure 9:**
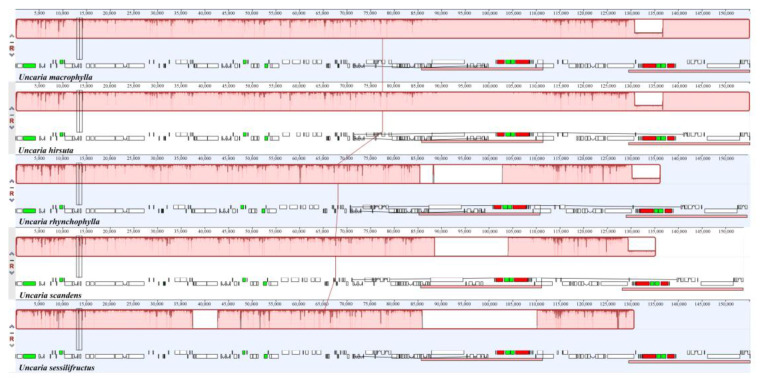
Synteny analyses for five *Uncaria* species in cp genomes.

The IR region is well-conserved, but the IR/SC boundary regions differ among plant species. The IR/SC border positions and their adjacent genes were compared using IRscope (Figure 8). In five cp genomes, the gene *rps19* crossed the LSC/IRb and was primarily found in the LSC region with a length of 247 bp and the remaining 32 bp in the IRb region. These overlapping sequences resulted in pseudogene sequences of *rps19* gene at the IRa/SSC boundary. The *ycf1* gene traversed the SSC and IRa regions, which was located at the SSC region with 4489–4504 bp (*U. sessilifructus*: 4504 bp, other: 4489 bp), while 1136 bp sequences located at IRa. These overlapping sequences resulted in the pseudogene sequences of *ycf1* gene at the IRb/SSC boundary. The *ndhF* genes did not cross the IRb/SSC boundary. JLA was located between *trnH* and *rpl2*, the distances between *trnH* and JLA were 9–12 bp. The results indicated that there were differences at the boundaries in the five cp genomes. Overall, the length variance of the complete genome sequences among the five cp genomes was caused by the contraction and extension of IR/SC boundary regions. In addition, the results of the synteny analysis showed that the chloroplast genomes did not undergo genomic rearrangements (Figure 9).

### 2.8. RNA Editing Site Analysis and Selective Pressure Analyses

The five species were predicted to have identical RNA editing sites. A total of 123 RNA editing sites were predicted in 28 genes (Supplementary Tables S4 and S5). All predicted editing sites were C to U transitions. The majority of predicted editing sites were found in the *ndhB* gene (14), which is consistent with the results for other plants, such as *Rhynchanthus beesianus, Pommereschea lackneri,* and *Hedychium coronarium* [20]. In addition, *psbB* contained ten predicted editing sites; *atpB* and *ndhD* contained nine predicted editing sites; *ndhF* contained eight predicted editing sites; and *atpA, psaB,* and *rpoB* contained seven predicted editing sites. From one to five predicted editing sites were present in the other gene (*rpoC2,* 5; *ndhA*, *atpI, ccsA, matK*, *ndhG, rpoA,* 4; *petB, petG,* 3; *accD, atpF, petD, rpoC1, rps14, rpl2,* 2; *psbE, psbF, rpl23, rps2, ycf3,* 1). Of the total number of RNA editing sites, there were 49 (39.84%) RNA editing sites at the first base of the codon and 74 (60.16%) RNA editing sites at the second base of the codon. The transformation of amino acids included 53 hydrophilic amino acids to hydrophobic amino acids (S→L, S→F, H→Y, T→M, R→W, T→I), 15 hydrophobic amino acids to hydrophilic amino acids (P→S), and 55 hydrophobic amino acids to different hydrophobic amino acids (P→L, P→F, L→F, A→V).

**Figure 10:**
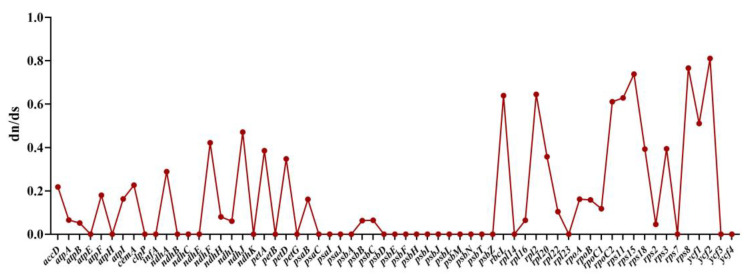
The dN/dS ratio values of common protein-coding genes in five *Uncaria* species chloroplast genomes.

**Figure 11:**
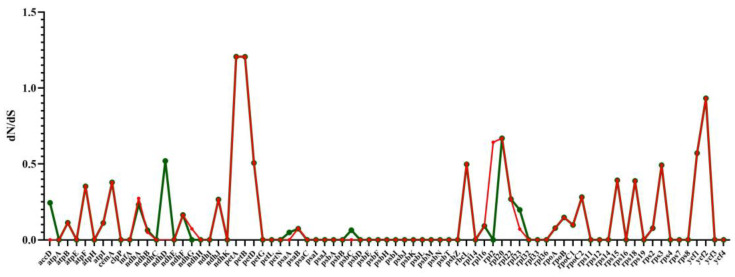
The dN/dS ratio values of common protein-coding genes in *U. macrophylla*, *U. hirsuta,* and *U. rhynchophylla*.

We calculated the ratio of dN/dS to discover proteins undergoing selection in the protein-coding genes. The ratio of ω = dN/dS has become a measure of selection pressure, with ω = 1, >1, and <1 indicating neutral evolution, positive selection, and negative or purifying selection, respectively [21]. The dN/dS were between 0.0001–0.81066 in five *Uncaria* species chloroplast genomes, the dN/dS values are all less than 1, suggesting that almost all of the genes were undergoing strong purifying selection. The dN/dS values of ten genes, including photosynthetic device genes and ribosomal protein genes, such as *ndhF*, *ndhJ*, *rbcL*, *rpl2*, *rpoC2*, *rps11, rps15, rps8, ycf1,* and *ycf2*, were >0.4, demonstrating that they are evolving particularly fast at the protein level. It can be inferred that there may be the potential for positive selection loci (Figure 10) [22]. Furthermore, most of the dN/dS ratio values in the protein-coding genes of *U. hirsuta* vs. *U. rhynchophylla* and *U. macrophylla* vs. *U. rhynchophylla* were less than 1, except *petA* and *petB*, whose values were 1.207 and 1.206, respectively, indicating that both genes were undergoing positive selection. In the protein-coding genes of *U. macrophylla* vs. *U. rhynchophylla* and *U. hirsuta* vs. *U. rhynchophylla,* most genes had the lowest dN/dS values. Meanwhile, we found seven genes (*ndhD, rbcL, petD, rpl20, rps3, ycf1, ycf2*) (dN/dS > 0.4) had the higher ratios of dN and dS in the *U. macrophylla* vs. *U. rhynchophylla* set, while seven genes (*petD, rbcL, rpl2, rpl20, rps3, ycf1, ycf2*) were also identified with higher dN/dS values in the *U. macrophylla* vs. *U. rhynchophylla* set (Figure 11).

### 2.9. Sequence Divergence Analysis

**Figure 12:**
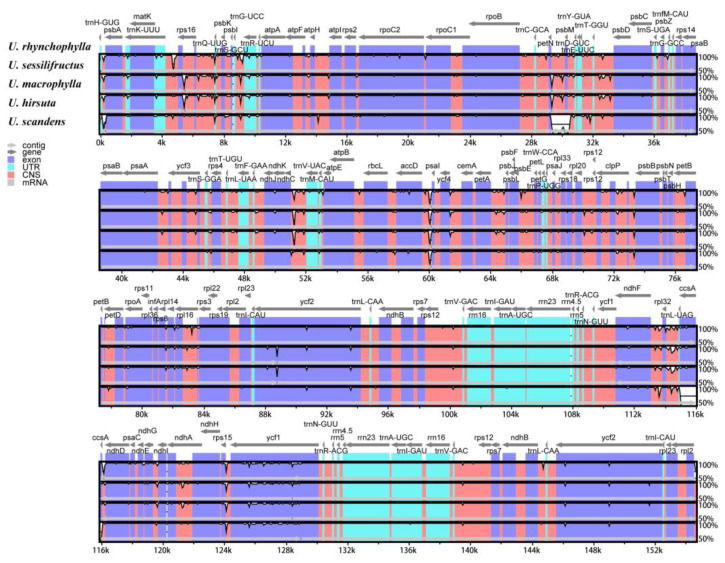
Sequence identity plots among five *Uncaria* species. The x-axis represents aligned base sequences, and the y-axis represents percent pairwise identity within 50–100%. (Coding regions were shown in blue, and non-coding regions were shown in pink. The locations and directions of each gene were indicated by gray arrows above the alignment. CNS: conserved noncoding sequences; UTR: untranslated region.).

**Figure 13:**
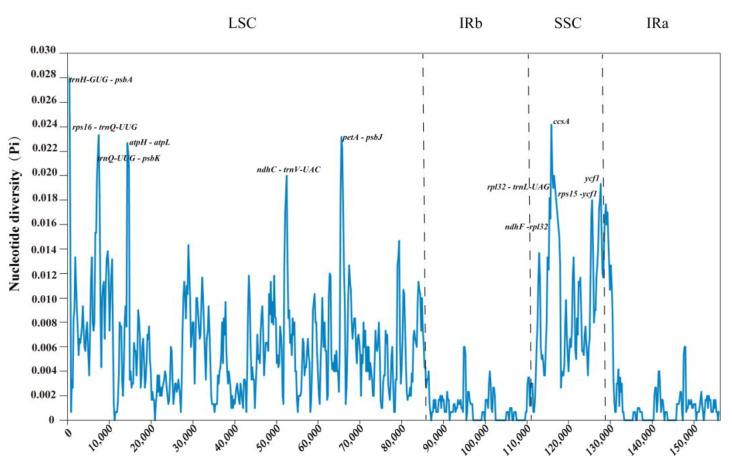
The nucleotide variability (Pi) values were compared among five *Uncaria* species. Position of the window midpoint on the X-axis and the nucleotide diversity within each window on the Y-axis (window length: 600 bp, step size: 200 bp).

Using *Uncaria rhynchophylla* (MN723865) as a reference, we used the mVISTA tool to compare the variations in five cp genomes and used DnaSP to identify the divergence hotspot regions (Figure 12 and Figure 13). The results revealed that the five species were proven to have high sequence identity by mVISTA. The pi value of nucleotide diversity ranged from 0 to 0.028. We found that nine intergenic spacers (IGSs) had relatively higher divergence values (Pi > 0.015) [23], including *trnH-GUG-psbA, rps16-trnQ-UUG, trnQ-UUG-psbK, atpH-atpL, ndhC-trnV-UAC, petA-psbJ, rpl32-trnL-UAG, ndhF-rpl32,* and *rps15-ycf1.* The partial sequences of the *ycf1* and *ccsA* genes showed a comparatively high nucleotide diversity. Most of the higher divergence regions were found in LSC and SSC regions, while IR regions showed a lower sequence divergence in the cp genomes. The results from mVISTA were consistent with those from DnaSP. The higher divergence regions can be used to identify the divergence between closely related species.

### 2.10. Phylogenetic Analysis

**Figure 14:**
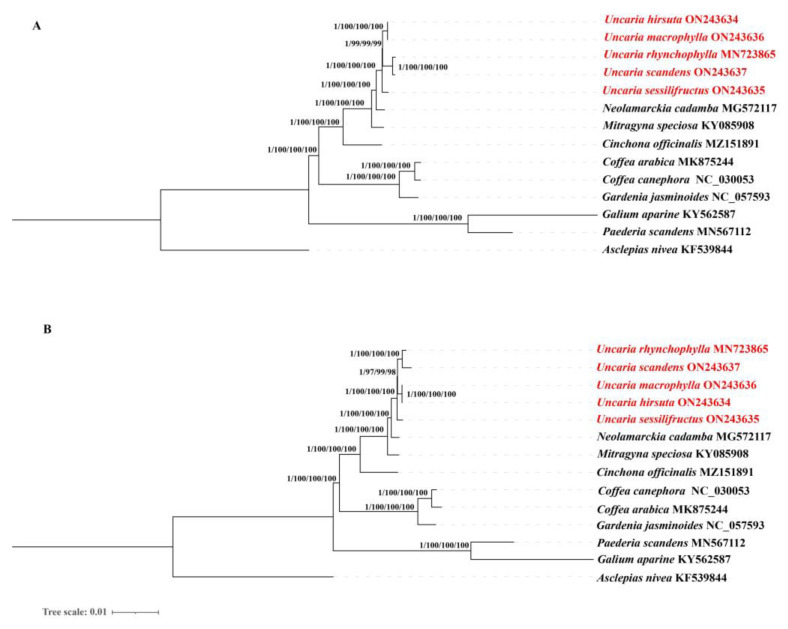
Phylogenetic trees based on 14 complete chloroplast genomes (**A**) and protein-coding genes (**B**) using different methods. From left to right, numbers above the branches are, respectively, represented Bayesian inference (BI) posterior probabilities (PPs) values, bootstrap support values of maximum likelihood (ML), neighbor-joining (NJ), and maximum parsimony (MP).

The cp genomes and protein-coding genes have been successfully used to reveal phylogenetic relationships [24,25]. The available chloroplast genomes sequences of 13 species from Rubiaceae and *Asclepias nivea* were selected to accomplish the phylogenetic analysis, including five *Uncaria* species, *Neolamarckia cadamba*, *Mitragyna speciosa*, *Cinchona officinalis, Galium aparine, Gardenia jasminoides, Paederia scandens, Coffea canephora,* and *Coffea arabica*. *Asclepias nivea* was set as outgroups. The bayesian inference (BI), maximum likelihood (ML), maximum parsimony (MP), and neighbor-joining (NJ) methods yielded nearly identical the tree topologies. Consequently, phylogenetic trees using four methods were integrated. From left to right, numbers above the branches are, respectively, represented Bayesian inference (BI) posterior probabilities (PPs) values, bootstrap support values of maximum likelihood (ML), neighbor-joining (NJ), and maximum parsimony (MP) from the complete cp genomes (Figure 14). The cp genome and protein-coding genes both produced phylogenetic trees with identical topological patterns. The phylogenetic trees all achieved relatively higher bootstrap support values and high BI (PP_BI_ = 1.00), nearly all bootstrap values reached 100%. A strong monophyletic branch of five *Uncaria* species was formed, and they are clustered into two clades with 100% bootstrap values and one PPs values. One monophyletic clade was included two sub-clades with 97–100% bootstrap values and one PPs values: *U. scandens, U. macrophylla,* and *U. rhynchophylla*, as well as *U. hirsuta*. *U. scandens* shares a clade with *U. rhynchophylla* and *U. macrophylla* shares a clade with *U. hirsuta*, which indicated that *U. scandens* was closely related to *U. rhynchophylla.* It is suggested that *U. hirsuta* and *U. macrophylla* were closely related. The other monophyletic clade included *U. sessilifructus* and four varieties of *Uncaria*, and the phylogenetic tree revealed that *U. sessilifructus* was closely related to four other varieties of *Uncaria* in this study.

## 3. Discussion

Currently, only a few numbers of the whole chloroplast genome sequences for the *Uncaria* genus were released into GenBank. Thus, the complete cp genomes of five *Uncaria* species were reported in this study. The cp genomes of five *Uncaria* species comprised 128–131 genes, including 83-86 protein-coding genes, 37 rRNA genes, and eight tRNA genes, other Rubiaceae plants have comparable gene patterns as well [26]. Chloroplast genomes are largely preserved in terms of gene composition and structure, compared to the nuclear and mitochondrial genomes. Additionally, recent research has demonstrated that gene or intron loss occurs in chloroplast genomes [27,28,29]. In addition, the deletion of the *petD* intron was observed in *U. macrophylla* and *U. hirsuta*. *petD* intron loss had been reported in numerous angiosperms, such as a few species of the families *Quercus* and *Brassicaceae* [30,31]. Introns play a key role in gene expression regulation. They can enhance gene expression in a given location and at a specific time [32]. Some introns have been shown to improve or necessitate proper amounts of mRNA transcription, processing, and transport. Intron loss appears to be under selection pressure in several unicellular eukaryotes [33]. The loss of the *ndhD* gene was only found in *U. hirsuta,* consistent with the cp genome of *Vigna angularis*, *Lathyrus sativus, Trifolium subterraneum,* and *Robinia pseudoacacia* [34]. Natural selection or chromosomal rearrangements during evolution may account for their absence in the species [35]. Pseudogenization and gene deletions were common and lineage-specific, with the *ndh* genes involved in electron recycling being particularly often and independently lost [29]. It is suggested that the *ndh* pathway is redundant in some species of *Uncaria* spp., and it may even be selected against [36]. Further investigation into the nature of cp genome modification should yield a new understanding of which genetic (and thus physiological) functions are lost. Thus, we infer that the deletion of some genes encoded in chloroplasts might not have an impact on the plants’ life cycle and homologous genes from other sources may be capable of performing the functions of deleted genes, although experiments are still needed to support this hypothesis.

The herbal medicine industry is rapidly expanding in China and the authenticity of herbs are becoming a major concern. *U. scandens* is a confusing counterfeit of *U. rhynchophylla,* so we sought to find the differences between these two species at the molecular level. Compared to the other four *Uncaria* species, two genes (*matk* and *ccsA*) are missing in *U. scandens.* The *ccsA* gene produces a protein called cytochrome C biogenesis, which mediates the binding of heme to c-type cytochromes [37,38]. *Pedicularis cyathophylloides* and *Pedicularis superba* also had *ccsA* gene deletion [39]. It is suggested that changes in the selection pressure on CCSA genes located at the junction of the IR and SSC regions may cause more frequent parallel loss or reversal of these genes relative to other genes in the LSC and IR regions. According to previous reports, the *matK* gene is a highly variable sequence frequently employed in many plant taxa phylogenetic studies [40,41]. Additionally, the loss of *matK* genes also occurs in the *Cuscuta* genus, *Arachnitis* genus, and *Thismia* genus [10,42]. The loss of *ccsA* and *matK* happened in other *U. scandens* individuals, which we had also uploaded on NCBI (GenBank ON086916). The current study will contribute to the identifying of *U. rhynchophylla* and its counterfeits, providing a specific experimental foundation for future studies.

The chloroplast of five *Uncaria* species was 153,780–155,177 bp with a quadripartite structure (LSC, SSC, and two IR regions), a characteristic of higher plants [43]. Whether in LSC regions, SSC regions, IR regions, or the complete cp genome, the five cp genomes of the *Uncaria* displayed a high degree of conservation in terms of GC content. The GC content was identical to the other Rubiaceae family members [26,44,45]. The IR areas contained four rRNA genes with high GC content, which supported the hypothesis of previous research [46,47,48,49]. A previous study found that the GC content was substantially higher, most likely due to the abundance of rRNA in the IR regions [50]. However, the exact specifics are still unknown. GC content is an important property of plastid genomes, which are likely produced following endosymbiosis by DNA replication and repair [51]. In viruses, GC content is not determined by the structure of the gene, but it is connected to its location [52]. More research is needed to determine if or not this exists in the chloroplast genome. It is supposed that one of the factors contributing to the low diversity in tRNA sequences and IR regions may be the high GC content, the maintenance of sequence stability may be greatly influenced by GC content. More evidence is needed from subsequent experiments to provide further.

During the evolution of terrestrial plants, the chloroplast genome’s IR region frequently experiences length variations, which results in the formation of a variety of boundary features [32,49,53]. Furthermore, the primary causes of the size change in cp genomes are the contraction and expansion of IR regions and the single-copy (SC) border regions, which influences the cp genome’s rate of evolution [54,55]. This research proved that boundary genes in the *Uncaria* species were mainly *rpl22, rps19, rpl2, ndhF, trnH,* and *ycf1*. Contraction and expansion as well as variation at the SC-IR boundary were identified, suggesting that gene location information in the SC-IR region can reveal the distance between species to some extent. During the evolution of terrestrial plants, length variation in the IR region of the chloroplast genome was a frequent occurrence, which resulted in the formation of a variety of boundary features [32,49,53]. Furthermore, the contraction and expansion of IR regions and the single-copy (SC) border regions are also the main contributors to the size variation in cp genomes, which influences the evolutionary rate of the cp genome [54,55]. This study demonstrated that boundary genes in the species of the *Uncaria* were mainly *rpl22, rps19, rpl2, ndhF, trnH,* and *ycf1*. Variation at the SC-IR boundary and contraction and expansion were observed in five *Uncaria* species, which suggested that the position information of the genes in the SC-IR region can, to some extent, reveal the distance between species.

It is a well-known fact that bias in codon usage occurs in wide different varieties of organisms. Codon usage preferences are closely linked to gene expression and have an impact on the level of mRNA and proteins in the genome [56,57,58,59]. The most abundant amino acid in the *Uncaria* was Leucine (Leu) with an average number of 2585, which had also been regularly observed in the other angiosperms. Meanwhile, in accordance with results for the chloroplast genomes of other angiosperms, our research showed that the majority of codons ending in A/U have RSCU values higher than 1, which may have been caused by a bias in composition toward a high A/T ratio [60,61,62]. Further studies will provide a deeper understanding of *Uncaria*’s gene expression and molecular evolutionary mechanisms. In the five cp genomes, the largest similarities in codon usage were discovered. These findings may indicate that these five *Uncaria* species underwent comparable environmental pressure during their evolutionary process. Identifying the RNA editing sites is critical for understanding the proper translation process and gene mutations in the cp genome [63]. In our study, potential RNA-editing sites in five *Uncaria* species were explored using the PREP tool. Twenty-eight genes were found to include a total of 123 predicted RNA editing sites, with the highest number found in the *ndhB* genes, which is consistent with the general features of RNA editing of chloroplast genes in higher plants [64].

The plastid genome contains many oligonucleotide repeats, which are assumed to be biomarkers for mutational hotspots [65,66,67,68]. In the present study, we detected forward repeats (F), reverse repeats (R), palindromic repeats (P), and complement repeats (C). Most of the dispersed repeated sequences were less than or equal to 30 bp. Tandem repeated sequences with periods ranging from 11 to 30 bp predominated in the cp genomes. The sequence length of tandem repeats of five *Uncaria* species ranged from 25 to 98 bp, which were similar to those found in *Salvia* [16]. In higher plants, SSRs are recognized as significant molecular markers for population variation investigations and are widely used to assess genetic diversity, population structure and evolutionary studies [69,70,71,72]. In addition, due to their distinct uniparental inheritance, SSRs have been widely used as molecular markers [73]. In total, five *Uncaria* species cp genomes with 31–49 perfect SSRs were found, with strong A/T bias. The majority of SSR types were discovered to be mono-nucleotide repeats in this study, which also discovered that the non-coding region included the largest number of SSRs. These repeats provide a crucial starting point for creating genetic markers in *Uncaria* species, which may be used in phylogenetic and ecological research. We will be able to assess the variation at the intraspecific level after the SSRs has been found in the cp genome of five *Uncaria* species.

In the current study, we identified that nine intergenic spacers (IGSs) had relatively high divergence values (Pi > 0.015), including *trnH-GUG-psbA, rps16-trnQ-UUG, trnQ-UUG-psbK, atpH-atpL, ndhC-trnV-UAC, petA-psbJ, rpl32-trnL-UAG, ndhF-rpl32,* and *rps15-ycf1.* Moreover, the partial sequences of the *ccsA* and *ycf1* genes showed a relatively high nucleotide diversity. These variable regions could be exploited to provide molecular markers for DNA barcoding and genealogical research in *Uncaria*. Over the last decade, nearly all taxonomic levels of phylogenetic connections have been successfully resolved using whole chloroplast genomes and protein-coding genes [24,25,74]. Our research provides the first phylogeny of the *Uncaria* genus based on a significant sampling of both chloroplast genomes and protein-coding genes. The topology structures of phylogenetic trees constructed with the whole chloroplast genome and protein-coding regions were almost identical. Phylogenetic analysis strongly demonstrated that *U. scandens* had been resolved as a sister relationship with *U. rhynchophylla*, and the same relationship was determined between *U. hirsuta,* and *U. macrophylla*. Furthermore, *U. sessilifructus* formed a clade with other four species. It is believed that *U. scandens* and *U. rhynchophylla* are more closely related because *U. scandens* is a commonly confused counterfeit of *U. rhynchophylla*. However, the results of the dN/dS analysis indicated that several different genes had the highest ratios of dN and dS, indicating that they were selected for sequence diversity and affect the sequence evolution rate of the chloroplast genomes of *U. hirsuta* and *U. machophylla*. This research will not only help shed lighter on the evolutionary position of *Uncaria* but also offer useful chloroplast genomic data for future investigations into the genesis and diversification of Rubiaceae. Overall, our phylogenomic investigations using chloroplast genomes have made the first successful effort to clarify intrageneric connections within the *Uncaria* genus [49].

## 4. Materials and Methods

### 4.1. Phenotype Measurement

The morphological characteristics of five *Uncaria* species were observed according to the taxonomic characteristics of botany and the characteristics of *Uncaria* spp. in Chinese flora. All the morphologic records based on our field observations.

### 4.2. Chloroplast DNA Extraction, Sequencing, Assembly and Annotation

To characterize the feature of among endemic species of *Uncaria* in China, five members of the *Uncaria* family were collected from Guangxi Province in China in July 2021 and subsequently deposited in the College of Life Sciences and Medicine, Zhejiang Sci-Tech University (Hangzhou, China), with herbarium numbers ZSTU70128 (*Uncaria rhynchophylla*), ZSTU09554 (*Uncaria macrophylla*), ZSTU01022 (*Uncaria hirsuta*), ZSTU09751 (*Uncaria sessilifructus*), and ZSTU70322 (*Uncaria scandens*). Following the manufacturer’s directions, DNA Plantzol Reagent (Invitrogen, Carlsbad, CA, USA) was used to extract the total DNA from the plant’s leaves [75,76,77]. Utilizing Illumina’s TruSeq Nano DNA Library Preparation kit (350 bp median insert), sequencing libraries were created in accordance with the manufacturer’s instructions [78,79,80]. The Illumina HiSeq 2500 platform was used to produce the chloroplast sequences (Illumina Inc., San Diego, CA, USA). Trimmomatic v0.39 software (Jülich, Germany) was used to treat all raw readings in order to eliminate adaptor sequences, short reads (length 75 bp), and low-quality bases (Q-value 20). Following that, adaptors were cut to produce high-quality clean reads (150 bp PE read length). To complete the chloroplast genome, these clear data were assembled utilizing GetOrganelle (-R 15-k 105,121) (Kunming, China) [81]. The sequence of *Uncaria rhynchophylla* (MN723865) was used as seeds for reference. Plastid-homologous sequences were selected from the total cellular DNA reads, and then assembled with a de novo assembly pipeline to obtain many longer fragment sequences (Contigs). The Contigs are then positioned on the chloroplast reference genomes of closely related species to obtain longer chloroplast genome fragment sequences. Finally, the full-length framework of the chloroplast genome sequence was constructed. The chloroplast genomes annotation was assembled based on the comparison by CPGAVAS2 (Beijing, China) [82] and downloaded the publicly available chloroplast genome of *Uncaria rhynchophylla* (GenBank accession number MN723865) from NCBI (Bethesda, MD, USA) as a reference for annotation. The annotation results were checked and corrected manually by Geneious v11.0.5 software (Biomatters, Auckland, New Zealand) [83]. The structural characteristics of the cp genomes were demonstrated using the CPGview-RSG (Beijing, China).

### 4.3. Codon Usage Analyses

The genetic code is degenerate, that means one amino acid of multiple codons is called the synonymous codons of this amino acid [84]. In different conditions, the synonymous codons selected by the amino acids on the protein during translation are not the same, and they prefer to select some specific codons for different species [85]. The selection phenomenon is called codon preference [86]. CodonW (Nottingham, UK) software was used to calculate the relative synonymous codon usage (RSCU).

### 4.4. IR/SC Boundary Region

Angiosperm chloroplast genomes’ length variation is commonly due to the expansion and contraction of the IR regions and the single-copy boundary regions [87,88]. We used the IRscope (Helsinki, Finland) (https://irscope.shinyapps.io/irapp/, accessed on 20 July 2022) to compare IR/SC boundary region and Mauve (Madison, WI, USA) [89] to compare the chloroplast genomes for synteny analyses.

### 4.5. Characterization of Repeat Sequences and SSRs

We used the REPuter (Bielefeld, Germany) (https://bibiserv.cebitec.uni-bielefeld.de/reputer, accessed on 20 July 2022) to identify dispersed repeats with the cp genome, including forward repeats, reverse repeats, palindromic repeats, and complement repeats [90]. The following settings were used: (1) Minimal Repeat Size of 8 bp; (2) Hamming Distance of 3; (3) Maximum Computed Repeats of 50; and (4) 90% or greater sequence identity. To explore tandem repeats with the cp genome using Tandem Repeats Finder (New York, NY, USA) (https://tandem.bu.edu/trf/trf.html, accessed on 20 July 2022) [91]. The parameter settings were used: (1) Match of 2; (2) Mismatch of 7; (3) Indel of 7; (4) Alignment score of 50; and (5) Maximum period size of 500. Simple sequence repeats (SSRs) in the cp genome were found using MISA (Seeland, Germany) (http://pgrc.ipk-gatersleben.de/misa/, accessed on 20 July 2022) [92], with the parameters set at ten repeat units ≥10 for mononucleotides, eight repeat units ≥8 for dinucleotides, four repeat units ≥4 for trinucleotides, and three repeat units ≥3 for tetranucleotides, pentanucleotide, and hexanucleotide.

### 4.6. Predict RNA Editing Sites and Selective Pressure Analyses

The alteration of nucleotides at the RNA level by RNA editing is frequent in higher organisms [93]. The nucleotide substitutions, deletions, or insertions occur in mRNA after gene transcription, resulting in a change in the original genetic information [94,95]. According to previous research, chloroplast RNA editing controls the genes’ expression, allowing the genes to produce a variety of protein products and increase genetic information [96]. In order to ensure the accuracy of the predictions, PREP-cp (Lincoln, NE, USA) (http://prep.unl.edu/, accessed on 20 July 2022) [97] was used to predict RNA editing sites, using a parameter threshold (cut-off value) of 0.8. To calculate the rates of synonymous ratio (dS) and nonsynonymous ratio (dN) substitution and their ratio (ω = dN/dS), the protein-coding sequences of five *Uncaria* species were aligned using the MAFFT v7.3 [98]. Then, stop codons and gaps between the comparative sequences were deleted. dS/dN ratios were finally calculated using the CODEML program in PAML (London, UK) [99].

### 4.7. Sequence Divergence Analysis

The genome comparison among the five *Uncaria* species in the chloroplast genomes was analyzed using the mVISTA program (Berkeley, CA, USA) (https://genome.lbl.gov/vista/mvista/submit.shtml, accessed on 20 July 2022) as a point of comparison. The Shuffle-LAGAN mode was used with default parameters to align the five chloroplast genomes [100]. The nucleotide diversity of coding sections, introns, and intergenic spacers was evaluated using DnaSP v6 software (Barcelona, Spain) to determine the divergence hotspot locations in the five cp genomes [101]. The regions mentioned above were selected based on two factors: the total number of mutations (Eta) was greater than zero, and the aligned length was more than 200 bp. The following settings for sliding window analysis were used: (1) windows length of 600 bp and (2) step size of 200 bp.

### 4.8. Phylogenetic Analysis

The available chloroplast genomes sequences and protein-coding genes of 13 species from Rubiaceae were selected to study for the phylogenetic analysis, including *Uncaria rhynchophylla, Uncaria sessilifructus, Uncaria macrophylla, Uncaria hirsuta, Uncaria scandens, Neolamarckia cadamba, Mitragyna speciosa, Cinchona officinalis, Coffea arabica, Coffea canephora, Gardenia jasminoides, Galium aparine,* and *Paederia scandens.* Using *Asclepias nivea* of *Asclepias* as an outgroup, the phylogenetic tree was built. Geneious v11.0.5 (Biomatters, Auckland, New Zealand) [83] software was used to extract protein-coding genes from the chloroplast genome. The alignment of chloroplast genomes sequences and protein-coding genes was generated by MAFFT v7.3 (Suita, Osaka, Japan) with the default parameters [98]. We employed four methods to structure phylogenetic trees. Bayesian inference (BI) analyses were conducted in PhyloSuite v1.2.2 software (Wuhan, China) [102] with the best-fit GTR+F+I+G4 model. The maximum likelihood (ML) phylogenetic analyses were performed using IQTREE v1.6.7 (Vienna, Austria) [103]. Utilizing the best TVM+F+R2 model with 5000 bootstrap replicates, the maximum likelihood (ML) phylogenetic analyses were executed using IQTREE v1.6.7 [103]. Maximum parsimony (MP) and Neighbor-joining (NJ) analyses used MEGA-X (State College, PA, USA) [104] with the 1000 bootstrap replicates.

## 5. Conclusions

The whole cp genomes of four *Uncaria* species were firstly revealed in present study. They are conserved by comparing the complete chloroplast genome of five *Uncaria*, but some highly variable loci in coding regions and intergenic regions occurred. Nine highly variable intergenic regions (*trnH-GUG-psbA, rps16-trnQ-UUG, trnQ-UUG-psbK, atpH-atpL, ndhC-trnV-UAC, petA-psbJ, rpl32-trnL-UAG, ndhF-rpl32,* and *rps15-ycf1*) and two highly variable genic regions (*ccsA* and *ycf1*) were detected, these regions could serve as significant molecular markers for the *Uncaria* genus. We also found that the *ndhB, psbB, ndhD,* and *atpB* genes are characterized by a large number of potential RNA editing sites and 31-49 SSRs in five *Uncaria* species cp genomes. Compared to *U. rhynchophylla,* the other four *Uncaria* species showed gene or intron loss. These results will provide a theoretical basis for identifying *U. rhynchophylla* and its counterfeits, as well as the species of the *Uncaria* genus. Phylogenetic analysis brought unprecedented perspectives on the relationship among five *Uncaria* species. The availability of these findings will supplement important genetic knowledge and serve as a helpful guide for future studies on plastid genetic engineering research in *Uncaria*.

## Figures and Tables

**Table 1:** Statistics of chloroplast genome sequencing of four species in *Uncaria*.

Sample	*U. sessilifructus*	*U. macrophylla*	*U. hirsuta*	*U. scandens*
Clean reads	18,093,602	18,325,401	14,436,561	13,659,533
Clean bases	2,300,585,478	2,759,589,867	2,174,504,818	2,031,770,495
Clean data	1.27 G	2.06 G	1.69 G	1.68 G
Read length/bp	150	150	150	150
Q30(%)	92.88%	95.21%	93.50%	92.58%
Length/bp	155,177	155,138	155,138	153,780
GenBank	ON243635	ON243636	ON243634	ON243637

**Table 2:** Comparison of chloroplast genomic characteristics among five *Uncaria* species.

	*U. rhynchophylla*	*U. sessilifructus*	*U. macrophylla*	*U. hirsuta*	*U. scandens*
**Length (bp)**					
Total	154,605	155,177	155,138	155,138	153,780
LSC	85,146(55.07%)	85,669(55.21%)	85,749(55.27%)	85,749(55.27%)	85,465(55.58%)
SSC	18,145(11.74%)	18,128(11.68%)	18,057(11.64%)	18,057(11.64%)	16,989(11.05%)
IR	25,657(16.60%)	25,690(16.56%)	25,666(16.54%)	25,666(16.54%)	25,663(16.69%)
CDS	80,517(52.08%)	80,027(51.57%)	80,069(51.61%)	77,390(49.88%)	77,526(50.41%)
tRNA	2789(1.80%)	2789(1.80%)	2789(1.80%)	2793(1.80%)	2717(1.77%)
rRNA	9048(5.85%)	9048(5.83%)	9048(5.83%)	9070(5.85%)	9048(5.88%)
**GC content (%)**					
Overall	37.7	37.6	37.5	37.5	37.7
LSC	35.6	35.5	35.4	35.5	35.5
SSC	31.7	31.5	31.5	31.7	31.7
IR	43.2	43.2	43.2	43.2	43.2
CDS	37.9	37.9	37.9	37.9	38.1
tRNA	53.3	53.5	53.5	53.5	53.4
rRNA	55.4	55.4	55.4	55.3	55.4
**Number of genes**					
Overall	131	130	130	130	128
CDS	86	85	85	85	83
tRNA	37	37	37	37	37
rRNA	8	8	8	8	8

## Data Availability

The chloroplast genome sequences of this study are openly available at the NCBI database (https://www.ncbi.nlm.nih.gov, accessed on 10 September 2022).

## Supplementary Materials

The following supporting information can be downloaded at: https://www.mdpi.com/article/10.3390/ijms231911617/s1.
