# Supplementary figures and images for: Characterization and Comparative Analysis of Chloroplast Genomes in Five Uncaria Species Endemic to China
Source: Int J Mol Sci. 2022 Oct 1;23(19):11617. doi: 10.3390/ijms231911617 (PMC9569570; doi:10.3390/ijms231911617)

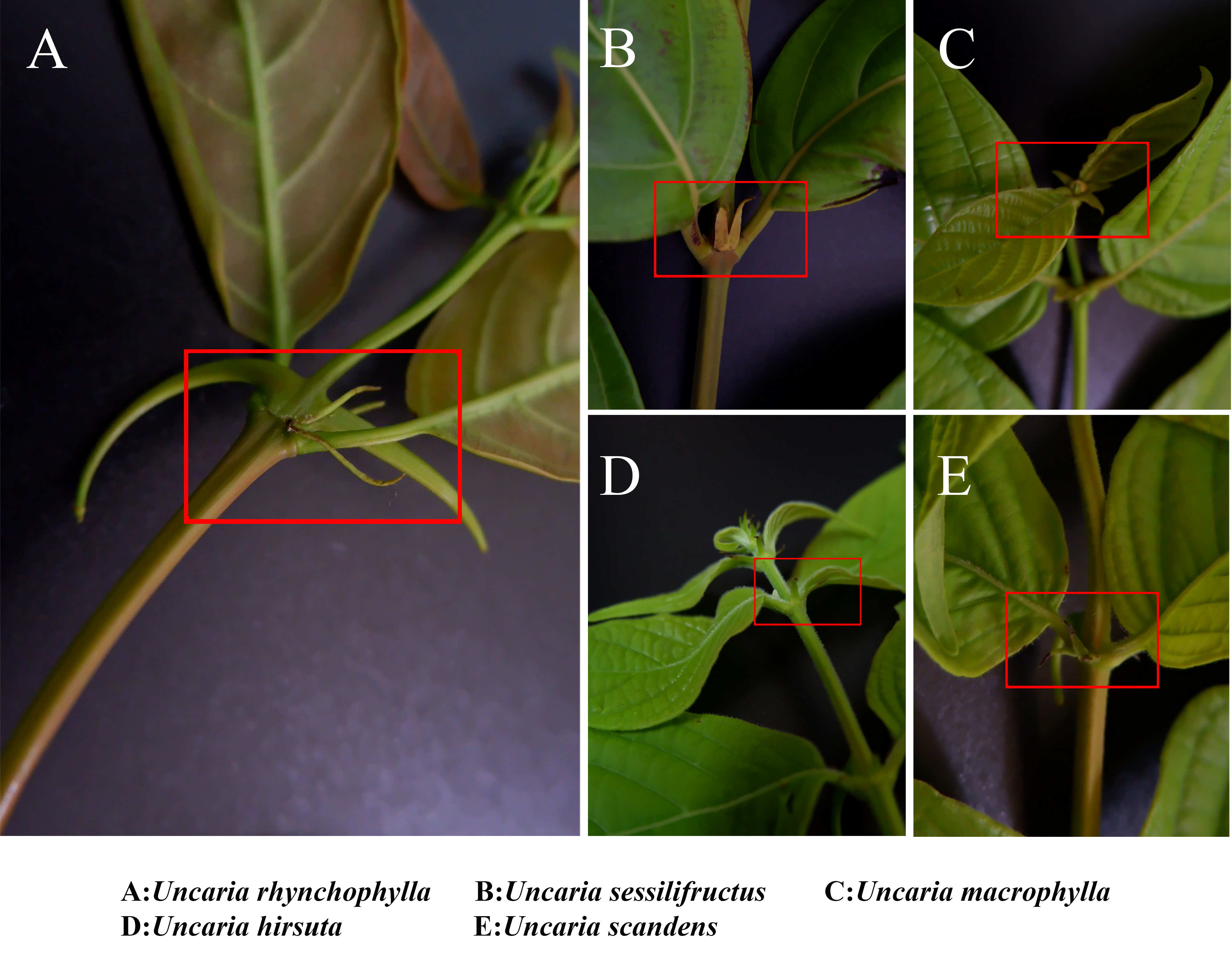

Supplement: Supplementary file 1 [file ijms-23-11617-s001.zip › Supplementary/Supplementary figure S1.tif]
